# Experiences of child abuse and prolonged grief in adulthood – results from a population-based study

**DOI:** 10.3389/fpsyt.2025.1606183

**Published:** 2025-09-15

**Authors:** Anna-Maria Rummel, Hannah Comtesse, Rita Rosner, Cedric Sachser, Jörg M. Fegert, Bettina K. Doering, Anna Vogel

**Affiliations:** ^1^ Department of Psychology, Catholic University Eichstaett-Ingolstadt, Eichstaett, Germany; ^2^ Department of Psychology, University of Hagen, Hagen, Germany; ^3^ Department of Clinical Child and Adolescent Psychology, Institute of Psychology, University of Bamberg, Bamberg, Germany; ^4^ Department of Child and Adolescent Psychiatry, Psychosomatics and Psychotherapy, University Medical Center Ulm, Ulm, Germany; ^5^ German Center for Mental Health (DZPG), Partner Site Ulm, Ulm, Germany; ^6^ Department of Psychology, Kiel University, Kiel, Germany

**Keywords:** childhood abuse, emotional abuse, physical abuse, sexual abuse, prolonged grief, PGD, cluster analysis, risk factors

## Abstract

**Background:**

In recent years, various risk factors for the development of prolonged grief disorder (PGD) have been discussed. While it is well established that child abuse increases the risk of various mental disorders in later life, the relationship between child abuse, including its subtypes, and PGD is barely examined.

**Objective:**

The aim of this study was to assess the impact of child abuse and distinct abuse patterns on PGD symptoms in a population-based German sample.

**Methods:**

We used self-reported data from 911 individuals (54.3 ± 17.9 years, 59.2% women) who had experienced the loss of a loved one. Participants completed demographic, loss-related and child abuse-related questions. To investigate PGD symptoms we utilized the Prolonged Grief Disorder-13+9 (PG13+9). The Childhood Trauma Questionnaire (CTQ) was employed to assess both overall child abuse severity as well as child abuse subtypes: child emotional abuse, child physical abuse, child sexual abuse. We used k-means cluster analysis to identify distinct child abuse patterns. Two hierarchical regression analyses were conducted to examine the associations between PGD symptom severity and child abuse and the identified child abuse clusters.

**Results:**

The prevalence rate for experiencing any child abuse was 13.5%. The occurrence of the child abuse subtypes was 7.9% for emotional abuse, 7.6% for physical abuse, and 5.9% for sexual abuse. *A priori*, we defined an “extreme abuse” cluster from the outliers and identified three clusters “no/low abuse”, “moderate abuse” and “high abuse” in our sample through the cluster analysis. Overall child abuse severity (*ß*=.13, *p*<0.001), “high abuse” cluster (*ß*=.12, *p*=0.001) and the *a priori* “extreme abuse” cluster (*ß*=.07, *p*=0.040) were significant predictors of PGD symptoms.

**Conclusion:**

Our study indicates that child abuse impacts PGD symptoms. Possible hypotheses for this connection and their implications are discussed.

## 1 Introduction

Child abuse is a global problem, over one billion children worldwide suffer some form of violence each year (1). The perception and definition of child abuse vary widely between countries, cultures, and families. For instance, in some places, corporal punishment is considered a disciplinary method and not a form of physical abuse (2). Prevalence rates of violence vary depending on the definition and measurement tool used (3, 4). Child emotional abuse encompasses both single incidents and ongoing failure by parents or caregivers to provide a supportive environment for the child’s development. This includes belittling, ridiculing, intimidating, threatening, and other non-physical forms of rejection or hostility (5). There are subtypes of emotional abuse, including childhood verbal abuse, which are sometimes studied separately (6). The prevalence rates for emotional abuse in non-clinical samples worldwide vary from 6.0 to 67.2%, with a median of 22.3% (range 6.0-51.2) for Europe (3). Child physical abuse is defined as non-accidental harm or the threat of harm to a child’s health, survival, development, or dignity. Physical abuse often occurs in the home environment as a disciplinary measure or punishment. It includes a range of behaviors, such as hitting, shaking, biting, choking, scalding, or poisoning that can vary in frequency and severity (5). In non-clinical samples, prevalence rates vary from 4.5% to 26.0%, with a median of 10.4% (range 4.7-15.2) for Europe (3). Child sexual abuse is defined as involving children in sexual activity that is beyond their developmental level, where they cannot make an informed decision, and that violates legal and social norms. Sexual abuse can be perpetrated by both adults and other children (5). It is the most extensively studied form of child abuse. In non-clinical samples, the prevalence of sexual abuse varies from 2.5% to 28.1% worldwide, with a median of 7.7% (range 5.7-8.8) in Europe (3). Many individuals who report child abuse also report neglect, which can occur in combination with other forms of child abuse and can be categorized as physical or emotional neglect (7). Child maltreatment encompasses both child abuse and child neglect. Neglect occurs when a caregiver is unable to provide a child with the necessary attention, care, or affection for healthy development due to indifference or personal impairment (8).

In a representative German study (7) conducted in 2016 with 2510 participants, 31.0% of the adult participants reported at least one subtype of child abuse or neglect. The prevalence rates were 6.5% for emotional abuse, 6.7% for physical abuse, and 7.6% for sexual abuse. The co-occurrence of several types of maltreatment was frequently reported (7). Participants who reported the highest rates of emotional abuse and physical abuse had higher risks of being unemployed, had a lower level of education and a lower income. Women were also more likely than men to report at least moderate sexual abuse and emotional abuse and older age was associated with higher rates in physical abuse (9). The prevalence rates of physical abuse and emotional abuse in Germany are below the European average, whereas the rates for sexual abuse were comparable (3, 7).

Child abuse is a significant health problem due to its association with various negative consequences for mental health in later life (10, 11). McKay et al. (12) reported a meta-analysis of longitudinal cohort studies on the association between child abuse and mental disorders. The results showed a significant association only for emotional abuse (odds ratio; OR=2.51), but not for sexual and physical abuse. Gardner et al. (13) conducted a meta-analysis of cohort and case-control studies to examine the association between child abuse and depression and anxiety: emotional abuse had the highest association with depression (OR=2.35), followed by sexual abuse (OR=2.11) and physical abuse (OR=1.78). The meta-analysis also found significant results for sexual abuse (OR=1.92) but not for physical abuse (OR=1.62) in relation to anxiety. However, the relationship between emotional abuse and anxiety was not examined (13). Experiencing multiple forms of child abuse increased the risk of developing mental disorders later in life (12, 13). These findings suggest that emotional abuse has the greatest impact on mental health in adulthood. Some authors attribute these findings (or lack of findings for physical abuse and sexual abuse) to inconsistencies in measurement instruments (12). However, among the available instruments for assessing child abuse, the Childhood Trauma Questionnaire (CTQ; 14) is a reliable instrument. It demonstrates good internal consistency and retest-reliability, and strong correspondence with interviews on child abuse (15). The CTQ comprises a total score as well as subscales for the various child abuse and neglect types. It is the most frequently utilized measurement instrument for assessing child abuse (16).

Given the tendency for different types of child abuse to co-occur, analyses that focus solely on one type of child abuse may be insufficient as they may underestimate the influence of other co-occuring child abuse types and their interactions (17). Alternatively, the severity or frequency of child abuse may be a more significant factor than the type (18). Therefore, several studies have aimed to identify patterns in individuals who experienced child abuse through the use of latent class or profile analyses, as well as hierarchical clustering analyses. Clustering methods are a popular technique for identifying empirical classifications in observations. There are several types of clustering, all of which are based on the fundamental principle of homogeneity (19). Therefore, clustering methods extend beyond the conventional boundaries of child abuse categorization, potentially providing new insights. Previous studies using the CTQ for clustering found inconsistent numbers of identified patterns (see **Table 1**). Most of these studies show a distinction according to intensity (17, 20–23). With the exception of the neglect pattern in Charak & Koots (22), these five studies exhibit comparable patterns that can be summarized as follows: no/low child abuse, moderate child abuse and high child abuse. Another study (24) identified analogous patterns, with the exception of a distinct pattern of sexual trauma. In addition to these findings, which appear to be largely consistent, Goerigk et al. (25, 26) identified numerous individual patterns, particularly in the no/low child abuse sector.

**Table 1:** Patterns identified in CTQ clustering studies.

Study	Nr. of patterns	Types of patterns
Begemann et al. (20)	3	Low, emotion-focused, multi-trauma
Schilling et al. (21)	3	Mild traumatization, multiple w/o sexual abuse, multiple w sexual abuse
Schilling et al. (17)	3	No/low, moderate, high child maltreatment
Wang et al. (22)	3	No/low, moderate w emotional abuse & neglect, high childhood trauma
Charak & Koot (23)	4	Neglect, low-moderate child abuse, moderate-severe child abuse, minimal child abuse or neglect
Utzinger et al. (24)	4	No/low, emotional, sexual, poly-trauma
Goerigk et al. (25)	7	No, minimal, predominant neglect (low), predominant neglect (high), neglect & sexual abuse, neglect & physical abuse, extreme traumatization
Goerigk et al. (26)	7	No, minimal, predominant emotional neglect & emotional abuse, predominant emotional neglect, predominant emotional neglect & emotional abuse, emotional neglect and abuse & physical abuse, emotional neglect and abuse & sexual abuse

CTQ, Childhood Trauma Questionnaire.

The impact of child abuse on health in adulthood appears to be influenced by various underlying aspects, including behavioral, emotional, social, and cognitive factors (27). Accordingly, it can be assumed that child abuse represents a vulnerability factor that can also influence how people deal with subsequent crises or adverse life events. However, it remains unclear at this point to what extent vulnerability is influenced by a specific, though very common, adverse life event: the loss of a loved one. Despite extensive research on child abuse and its impact on mental health in later life, the impact of child abuse on the development and maintenance of prolonged grief disorder (PGD) symptoms in adulthood remains largely unclear. Given that PGD has a cross-national average prevalence rate of 5% in probability samples (28), and a 1.5% prevalence rate in a representative sample of the German general population (29), it can be considered a relevant mental disorder. PGD is a recently introduced diagnosis in the ICD-11 (30) and the text revision of the DSM-5 (DSM-5-TR; 31). It is characterized by an enduring and intense yearning for or preoccupation with the deceased person, as well as intense emotional distress, disruption of identity, loss of meaning in life, and profound loneliness (32). Recent research has identified several risk factors. Although some demographic and grief-related factors, such as older age, unexpectedness of death, relationship to the deceased, and time since loss, are frequently reported as predictors, findings on gender, education, and employment status are more inconsistent (28, 33–39).

However, there is limited literature on the impact of childhood factors, such as child abuse, on the development of PGD in adulthood. Silverman et al. (40) interviewed 85 widowed individuals (aged 28–81 years) and asked an open-ended question about their experiences of child abuse. Only four participants (5.0%) reported experiencing abuse during childhood, with three reporting sexual abuse and one reporting verbal abuse and physical abuse. These four participants exhibited high levels of PGD symptoms, but the average time since loss was less than six months, which does not even meet the less stringent time criterion of ICD-11. Treml et al. (41) analyzed a large sample of elderly Germans (N=2,865) aged between 60–80 years. Child maltreatment, including abuse and neglect, was examined using the Childhood Trauma Screener (42), a shortened version of the CTQ. The Childhood Trauma Screener measures each subtype of child abuse and neglect with a single item, and a total score can be calculated. A higher score indicates a greater prevalence of child maltreatment. Treml et al. (41) found no significant association between child maltreatment and PGD symptoms. These studies’ results are limited due to partially small sample sizes, restricted age ranges, a lack of reference to current PGD criteria, and a lack of valid measurement instruments that allow for precise differentiation between different subtypes of child abuse or creating clusters.

Although the available data is limited and inconsistent, we anticipate a relationship between child abuse and PGD for the following reasons. Research has shown an increased likelihood of developing mental disorders later in life, at least after emotional abuse. It is assumed that this is also true for other types of child abuse, and that the reasons for no-findings are of a methodological nature (12). Additionally, research has shown that experiencing multiple types of abuse and neglect during childhood increases the likelihood of developing a mental disorder (11, 12) Furthermore, the frequency or severity of the abuse is a more important factor than the specific type (18). In this context, clustering may prove an efficacious method for integrating the acquired knowledge. The symptoms of PGD frequently manifest alongside other disorders as a complicated grief reaction (43). A meta-analysis indicates that the probability of co-occurrence with symptoms of depression is 63%, with symptoms of anxiety is 54%, and with symptoms of posttraumatic stress disorder (PTSD) is 49% (43). These disorders have a better-studied relationship with child abuse, and there is an elevated probability of occurrence for individuals who have experienced particularly emotional abuse (12, 13). Given the overlap between symptoms of depression and PTSD with PGD symptoms (44) and the relationship between child abuse and high PGD symptoms in a small study (40) a relationship between PGD symptoms and child abuse can be suspected.

The first aim of this study was to investigate the prevalence of different subtypes of child abuse (emotional, physical, and sexual) in a population-based German sample of individuals who reported the loss of a significant person. The second aim was to identify distinct patterns of child abuse in this sample. Finally, we aimed to examine the association between both the overall burden of child abuse, as well as different child abuse patterns, and PGD symptoms, while controlling for the influence of common associated factors (age, gender, marital status, time since loss, expectedness of death, relationship to the deceased).

## 2 Materials and methods

### 2.1 Ethics

The study was approved by the institutional review board of the University of Leipzig, Germany (145-19/ek, April, 2^nd^, 2019). All participants were informed about the purpose and procedures of the study before giving their written informed consent. This study was not pre-registered.

### 2.2 Participants and procedure

This cross-sectional observational study was conducted as part of a multitopic survey on the physical and mental well-being of the German population, conducted by a demographic consulting firm (USUMA GmbH, Berlin, Germany) from May to July 2019. To determine the sample, (a) the German territory was divided into 258 sample areas representing the entire country, (b) the household was selected using random route and walkthrough procedures, and (c) the person to be interviewed within the household was identified from those who met the inclusion criteria (≥14 years of age and sufficient knowledge of German) using Kish-selection technique. Out of the 5,393 valid household addresses contacted, data was not available for 2,851 individuals due to the following reasons:

Household declined participation (22.9%) or could not be reached after four visits (13.6%), target person declined participation (12.3%), could not be reached after four visits (3.0%), was absent (0.6%), or unable to follow the survey (0.5%). Sociodemographic data were collected from the remaining 2,542 subjects by trained interviewers after obtaining informed consent. Due to the sensitivity of the data, the participants completed self-report measures using paper-pencil versions. The trained interviewer was present during the self-assessment to provide support in the event of problems or questions. Of the 2,542 participants, 11 interviews could not be analyzed due to a high number of unanswered questions. Participants who reported the loss of a significant person were considered for the current study and completed an extended version of the Prolonged Grief Disorder-13 (PG13+9; 45). Out of the 2,531 participants, 1,584 did not report the death of a loved one, 33 did not complete the PG-13 despite reporting a significant loss, and 3 did not complete the CTQ. Therefore, the final sample included 911 participants. The majority of participants were female (59%) with a mean age of 54.3 ± 17.9 years. On average, the death of the significant other happened more than 8 years ago and more than 40% experienced the death as unexpected. For further demographic and loss-related information, please refer to **Table 2**. Of the 240 persons who reported abuse, 49.2% (*n*=69) identified the abuser as a family member, such as a (step)parent, sibling, uncle, aunt, or cousin. The second most commonly reported abuser category, at 21.8% (*n*=27), was someone known to the victim, such as a neighbor or family friend. Only 6.5% (*n*=8) reported not having previously known their abuser, and 22.6% (*n*=28) experienced abuse by persons in more than one category. Men were responsible for 7.6% (*n*=10) of the abuse, women for 10.6% (*n*=14), and both sexes for the majority of cases (81.8%, *n*=108). The sample was representative of the German micro census in terms of age, gender, and geographic region. The micro census is a survey that represents 1% of the German population, approximately 810,000 Germans, and is used for policy decision making in Germany (46).

**Table 2:** Demographic and loss-related characteristics (N=911).

Variables	*n/M*	*%/SD*
Demographic characteristics		
Gender, *n/%*		
Female	539	59.17
Male	372	40.83
Age in years, *M/SD*	54.32	17.99
Marital status *n/%*		
Married/long-term relationship	380	41.71
Single/divorced/separated	370	40.61
Widowed	158	17.34
No response	3	0.33
Education *n/%*		
Primary	24	2.63
Secondary	771	84.63
Tertiary	99	10.87
In school/other/no response	17	1.87
Employment status *n/%*		
Employed	448	49.18
Unemployed	33	3.62
Retired	330	43
In training/education	5.7	28
Other/no response	0.6	3
Citizenship *n/%*		
German	859	94.50
Other	43	4.73
German and other	7	0.77

Loss-related characteristics		
Time since loss in months, *M/SD*	105.12	115.23
Relationship with the deceased, *n/%*		
Child	32	3.51
Partner	159	17.45
Parent	372	40.83
Other family member	257	28.21
Friends	87	9.55
No response	4	0.44
Expectedness of the death, *n/%*		
Expected	426	46.76
Unexpected	388	42.59
None or both	94	10.32
No response	3	0.33

### 2.3 Measures

#### 2.3.1 Sociodemographic, loss- and child abuse-related information

Participants provided information on their age, gender, marital status, education, employment status, income, whether they live with a partner, and citizenship. Additionally, we collected information about their relationship with the deceased, the expectedness of the death, the time since the loss, and the gender and relationship to the abuser.

#### 2.3.2 Prolonged Grief Disorder 13 (PG13+9)

The PG13+9 (45) is an extended German version of the PG-13 (47) to assess PGD symptoms. The participants rated their symptoms on a 5-point scale according to their frequency (1 = ‘not at all’ to 5 = ‘several times a day’) or intensity (1 = ‘not at all’ to 5 = ‘overwhelmingly’). In the present study, only the original eleven 5-point items of the PG-13 score were analyzed. PGD symptom severity was calculated by summing these items (theoretical range of 11-55). The mean PG-13 score in our study was 19.2 ± 8.7 with an excellent internal consistency (*α*=.95).

#### 2.3.3 Childhood Trauma Questionnaire (CTQ)

Child abuse was assessed using the CTQ (14). The German version of the CTQ is a reliable and valid self-report instrument for the retrospective assessment of child maltreatment (48). In the present study, only items that investigated child abuse were utilized. The 15-item scale measures experiences of physical abuse, emotional abuse, and sexual abuse, with five items for each subscale. The occurrence of child abuse was rated by participants on a 5-point scale ranging from 1 (never) to 5 (very often), resulting in a possible score range of 15 to 75. Our study found good internal consistency (*α*=.93) for the total score, as well as good to excellent internal consistency for the subscales: *α*=.90 for emotional abuse, *α*=.88 for physical abuse, and *α*=.94 for sexual abuse. The CTQ assesses instances of child abuse and generates a total score, as well as scores for each of the three subtypes of child abuse. Häuser et al. (49) proposed a severity classification system based on the severity of child abuse experiences. The severity categories are determined by the sum score of the items for each subtype and can be classified as non-minimal (emotional abuse=5-8, physical abuse=5-7, sexual abuse=5), minimal-moderate (emotional abuse=9-12, physical abuse=8-9, sexual abuse=6-7), moderate-severe (emotional abuse=13-15, physical abuse=10-12, sexual abuse=8-12), and severe-extreme (emotional abuse=16-25, physical abuse=13-25, sexual abuse=13-25). Prevalence rates were calculated from all individuals who reported at least moderate-severe abuse in the different subtypes, following the recommendation of Witt et al. (9).

### 2.4 Statistical analyses

All analyses were conducted using Stata version 18.5 (50). A total of 18 cases were identified, in which a single item was missing in a CTQ category and in one case five items were missing. In these cases, the category and the total score were excluded from the subsequent analysis. To ascertain whether a cluster structure exists within the data set and to identify outliers, a single-linkage analysis was conducted (19). Cases that exhibited a notable degree of divergence from the remaining cases in the dendrogram were excluded from the cluster analysis. As k-means is highly susceptible to noise (19, 51) and the analysis of data containing outliers would compromise the quality of the clusters (52), we excluded them prior to clustering analysis. The proximity between individuals was determined using the Euclidean distance metric (53). The optimal number of clusters was determined based on evidence-based considerations of previous cluster analysis with the CTQ (e.g., 17, 20–22). In addition to a three-cluster solution, two-, four-, and five-cluster solutions were also examined. The determination of the optimal cluster solution for the data was based on the Calinski–Harabasz Index (54) and content-related considerations. The clustering model was tested several times, and the optimal solution was identified through the calculation of the root mean square error (55). The outliers were assigned to a separate cluster *a priori* in order to include them for further analysis. This approach aligns with the proposal of Gan and Ng (56), who proposed an additional outlier cluster to hold the outliers. To identify potential predictors, we analyzed the correlation between PGD symptom severity and various factors. For continuous variables, such as age, time since loss, and the CTQ total score, we used Pearson correlations. Nominal scale variables, such as gender, marital status, expectedness of the death, relationship with the deceased, and the identified child abuse-clusters, were dummy coded to calculate their correlation with the severity of PGD symptoms. The gender reference category was ‘female’, and the reference category for marital status was ‘not married or long-term relationship’. The reference category for the expectedness of death was ‘expected loss’, and for the relationship to the deceased it was ‘loss of a parent’. In terms of the child abuse clusters, the reference category was ‘no/low abuse’. To assess whether overall child abuse as the CTQ total score or child abuse clusters predict the severity of PGD symptoms, two hierarchical regression analyses were conducted. Both analyses included demographic variables such as age, gender, and marital status in the first step. The second step involved adding loss-related variables such as time since loss, relationship to the deceased, and expectedness of the death. The third step differed in terms of consideration of child abuse. In hierarchical regression analysis 1, the overall severity of child abuse was included. In the third step of hierarchical regression 2, the child abuse clusters were used instead. A significance level of *p*=.05 was used for all tests.

## 3 Results

### 3.1 Prevalence of child abuse and subtypes in bereaved adults

In our sample, the mean CTQ total score was 18.5 ± 6.9. A differentiated view of the types of child abuse results in a mean score of 7.0 ± 3.5 for the emotional abuse specific questions of the CTQ, for physical abuse 6.0 ± 2.5 and for sexual abuse 5.5 ± 2.2. According to Häuser et al. (49)’s severity categories, the majority of respondents experienced non-minimal child abuse, with percentages ranging from 79.8% to 90.2% depending on the subtype of child abuse. **Table 3** provides a detailed list of the subscales by severity classification. The prevalence rate of child abuse (at least moderate-severe level) was 13.5% (*n*=120) in our sample. The analysis of the different types of child abuse reveals a prevalence rate of 7.6% (*n*=69) for physical abuse, 7.9% (*n*=71) for emotional abuse, and 5.9% (*n*=52) for sexual abuse. For more information on the prevalence of child abuse, see **Table 4**.

**Table 3:** Child abuse by severity categories according to Häuser et al. (49).

CTQ	Emotional abuse (*N*=902)	Physical abuse (*N*=907)	Sexual abuse (*N*=905)
Severity category	*n* (%)	*n* (%)	*n* (%)
Non-minimal	720 (79.82)	792 (87.32)	816 (90.17)
Minimal-moderate	111 (12.31)	46 (5.07)	37 (4.09)
Moderate-severe	32 (3.55)	32 (3.53)	31 (3.43)
Severe-extreme	29 (4.32)	37 (4.08)	21 (2.32)

CTQ, childhood trauma questionnaire.

**Table 4:** Prevalence of child abuse (N=892).

Variables	*n*	*%*
Any child abuse	120	13.45
Types of child abuse		
Emotional abuse only	21	2.35
Physical abuse only	20	2.24
Sexual abuse only	25	2.80
Emotional + physical abuse	29	3.25
Emotional + sexual abuse	5	0.56
Physical + sexual abuse	6	0.67
Emotional + physical + sexual abuse	14	1.57

The prevalence rate is calculated from all who reported at least moderate-severe levels.

### 3.2 Cluster analysis

**Figure 1:**
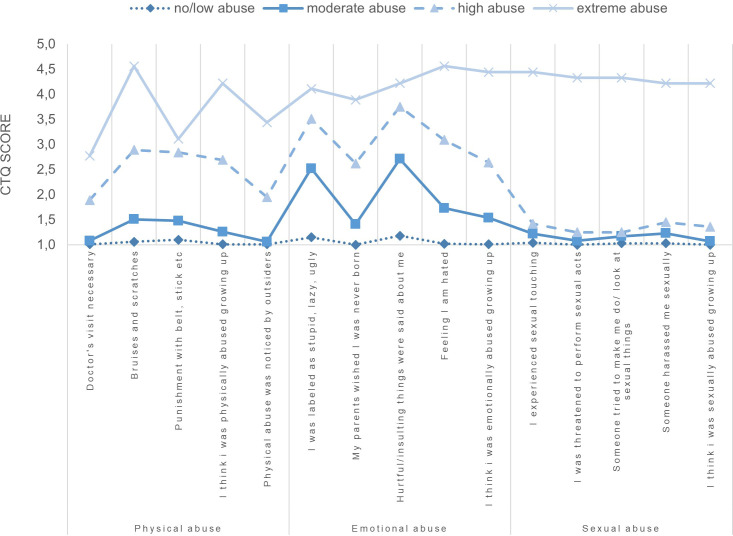
Mean symptom scores of the CTQ items per cluster.

Nine cases appeared significantly distant from the remaining 885 cases in the dendrogram and were therefore not included in the cluster analysis. A review of the nine cases that were removed revealed a high degree of similarity between them. Eight of these cases had experienced severe-extreme child abuse in all subtypes, while one case had experienced severe-extreme physical abuse and sexual abuse and moderate-severe emotional abuse. As these are extreme values that do not constitute erroneous outliers, and removing them would result in a loss of information, we have placed these cases *a priori* in a separate cluster for further analysis. For the remaining cases the k-means cluster-analysis yielded a three-cluster solution. The first cluster, labeled as “no/low abuse” comprised 666 participants. The second cluster, labeled “moderate abuse”, comprised 162 participants and the third cluster with 55 participants was labeled as “high abuse”. To assess the external cluster validity, we used the adjusted rand index (ARI; 57) and tested a three-cluster solution in the entire sample of 2,315 participants, including those who had not lost a loved one. The visual detection of the cluster structure revealed a high degree of similarity, with an ARI of .983, which indicated an excellent level of agreement. The *a priori* defined outlier cluster comprises nine participants and is labeled as “extreme abuse”. For the demographic and loss-related information of the individual clusters, see **Table 5**. No significant difference was found between the clusters for demographic and loss-related information. As expected, there is a significant mean difference between the clusters for the CTQ total score and all subtypes. These findings persisted in *post hoc* Bonferroni corrected analyses. **Figure 1** shows the mean scores for the CTQ items by cluster.

**Table 5:** Characteristics of the clusters.

Variables	No/low abuse (N=666)	Moderate abuse (N=162)	High abuse (N=55)	Extreme abuse (N=9)*	*F/Chi2 (p)*
Demographic characteristics					
Gender, *n (%)*					0.75 (0.860)
Female	390 (58.56)	100 (61.73)	31 (56.36)	5 (55.56)	
Male	276 (41.44)	62 (38.27)	24 (43.64)	4 (44.44)	
Age in years, *M* ± *SD*	54.65 ± 17.98	52.18 ± 18.25	56.45 ± 16.85	43.89 ± 14.46	2.11 (0.098)
Marital status *n (%)*					2.64 (0.451)
Married/long-term relationship	287 (43.29)	64 (39.51)	21 (38.18)	2 (22.22)	
Not married or long-term relationship	376 (56.71)	98 (60.49)	34 (61.82)	7 (77.78)	
Loss-related characteristics					
Time since loss in months, *M* ± *SD*	102.48 ± 115.57	102.21 ± 98.44	131.55 ± 151.04	127.5 ± 81.80	1.13 (0.335)
Relationship with the deceased, *n (%)*					13.91 (0.532)
Child	19 (2.85)	7 (4.32)	2 (3.64)	0	
Partner	123 (18.47)	23 (14.20)	8 (14.55)	1 (11.11)	
Parent	277 (41.59)	59 (36.42)	28 (50.91)	4 (44.44)	
Other family member	187 (28.08)	51 (31.48)	11 (20.00)	2 (22.22)	
Friends	56 (8.41)	22 (13.58)	6 (10.91)	2 (22.22)	
Expectedness of the death, *n (%)*					6.92 (0.075)
Expected	317 (52.83)	78 (54.55)	18 (37.50)	2 (25.00)	
Unexpected	283 (47.17)	65 (45.45)	30 (62.50)	6 (75.00)	
Child abuse related characteristics					
CTQ total score, *M* ± *SD*	15.65 ± 1.26	22.08 ± 3.32	34.6 ± 5.46	60.89 ± 5.40	2397.61 (<0.001)
Subtypes, *M* ± *SD*					
Emotional abuse	5.36 ± 0.68	9.93 ± 2.13	15.6 ± 3.38	21.22 ± 4.63	1466.69 (<0.001)
Physical abuse	5.19 ± 0.63	6.40 ± 1.89	12.25 ± 3.67	18.11 ± 2.32	724.22 (<0.001)
Sexual abuse	5.10 ± 0.70	5.76 ± 1.96	6.75 ± 3.09	21.56 ± 3.68	474.40 (<0.001)

PG-13, Prolonged Grief Disorder 13; CTQ, Childhood Trauma Questionnaire.*Extreme abuse cluster, *a priori* defined outlier cluster.

### 3.3 Associations between child abuse experiences and PGD symptoms

The results of the hierarchical regression analysis with PGD symptom severity and overall severity of child abuse are shown in **Table 6**. Step 1 included sociodemographic variables such as age, gender, and marital status which explained 3.0% of the variance. Gender (*ß*=.09) and marital status (*ß*=-.14) were significant contributors to this step. Step 2 introduced variables related to the loss, including time since loss, unexpectedness, and relationship to the deceased, and explained an additional 15.6% of the variance. Time since loss had the greatest impact among the loss-related variables. After entering the loss-related variables, marital status (*ß*=-.08), time since loss (*ß*=-.23), unexpectedness of death (*ß*=.22), loss of a child (*ß*=.20), and loss of a partner (*ß*=.19) were all found to be significant. In the third step, overall child abuse severity (CTQ total score) was added, which increased the explained variance to 20.3%. The final model showed that time since loss (*ß*=-.23), unexpectedness of death (*ß*=.21), loss of a child (*ß*=.21), loss of a partner (*ß*=.20), and overall child abuse severity (*ß*=.13) were significant contributors to the severity of grief symptoms. The full model [*F* (10,701) = 17.87, *p*<0.001] explained 20.3% of the variance in grief symptoms. A second hierarchical regression analysis was performed using the child abuse clusters identified instead of the overall severity of child abuse (see **Table 7**). The first and second steps were consistent with the previous analysis. In the third step, the cluster moderate abuse, high abuse and extreme abuse were entered, explaining an additional 1.8% of the variance. In the final model of the second analysis, the following variables significantly contributed to grief symptoms: time since loss (*ß*=-.23), unexpectedness of death (*ß*=.21), loss of child (*ß*=.21), loss of partner (*ß*=.20), *a priori* extreme abuse cluster (*ß*=.07) and high abuse cluster (*ß*=.12). The model accounted for 20.4% of the variance in grief symptoms [*F* (12,699) = 14.92, *p <*0.001]. In both hierarchical regression analyses, the adjusted R^2^ indicated a moderate effect size (19.0% and 19.2%) according to Cohen (58). No evidence of multicollinearity was found, as all VIF values were less than two. The standardized regression coefficients of the final models indicated that five or six variables significantly contributed to the severity of PGD symptoms. Consequently, it was demonstrated that, in addition to loss-related variables, child abuse exerts a significant influence on PGD symptoms.

**Table 6:** Hierarchical regression with the criterion PGD symptom severity and overall severity of child abuse.

	Demographic variables					Loss-related variables					Child abuse variable				
	*B*	*SEB*	*ß*	*t*	*p*	*B*	*SEB*	*ß*	*t*	*p*	*B*	*SEB*	*ß*	*t*	*p*
Age	0.03	0.02	.05	1.40	0.161	0.00	0.02	.01	0.14	0.888	0.00	0.02	.01	0.14	0.892
Gender	1.50	0.65	.09	2.31	0.021	1.17	0.61	.07	1.93	0.054	1.09	0.60	.06	1.81	0.071
Marital status	-2.42	0.64	-.14	-3.75	<0.001	-1.39	0.65	-.08	-2.13	0.034	-1.15	0.65	-.07	-1.77	0.077
Time since loss						-0.02	0.00	-.23	-6.35	<0.001	-0.02	0.00	-.23	-6.54	<0.001
Expectedness						3.71	0.61	.22	6.07	<0.001	3.56	0.61	.21	5.87	<0.001
Loss of child						10.34	1.78	.20	5.82	<0.001	10.66	1.76	.21	6.05	<0.001
Loss of partner						4.26	1.01	.19	4.22	<0.001	4.59	1.00	.20	4.58	<0.001
Loss of other family members						-0.28	0.76	-.01	-0.37	0.714	-0.17	0.75	-.01	-0.22	0.825
Loss of friend						0.31	1.07	.01	0.29	0.771	0.09	1.07	.00	0.09	0.930
Child abuse											0.17	.04	.13	3.88	<0.001
Model summary															
R^2^ (adj. R^2^)	.030 (.026)					.186 (.176)					.203 (.192)				
Δ*R* ^2^	.030^**^					.156^**^					.017^**^				

Gender, reference category: men; Marital status, reference category: not married or long-term relationship; Expectedness, reference category: expected death; Loss of a child/partner/other family member/friend, reference category: loss of parent; Child abuse measured with the total score of the Childhood Trauma Questionnaire.

^**^p <.01; ^*^p <.05.

**Table 7:** Hierarchical regression with the criterion PGD symptom severity and the clusters based on the Childhood Trauma Questionnaire.

	Demographic variables					Loss related variables					Child abuse variables				
	*B*	*SEB*	*ß*	*t*	*p*	*B*	*SEB*	*ß*	*t*	*p*	*B*	*SEB*	*ß*	*t*	*p*
Age	0.03	0.02	.05	1.40	0.161	0.00	0.02	.01	0.14	0.888	0.00	0.02	.00	0.13	0.939
Gender	1.50	0.65	.09	2.31	0.021	1.17	0.61	.07	1.93	0.054	1.11	0.60	.06	1.85	0.084
Marital status	-2.42	0.64	-.14	-3.75	<0.001	-1.39	0.65	-.08	-2.13	0.034	-1.17	0.65	-.07	-1.80	0.065
Time since loss						-0.02	0.00	-.23	-6.35	<0.001	-0.02	0.00	-.23	-6.45	<0.001
Expectedness						3.71	0.61	.22	6.07	<0.001	3.54	0.61	.21	5.82	<0.001
Loss of child						10.34	1.78	.20	5.82	<0.001	10.67	1.77	.21	6.03	<0.001
Loss of partner						4.26	1.01	.19	4.22	<0.001	4.59	1.00	.20	4.58	<0.001
Loss of other family members						-0.28	0.76	-.01	-0.37	0.714	-0.11	0.76	-.01	-0.15	0.996
Loss of friend						0.31	1.07	.01	0.29	0.771	0.21	1.07	.01	0.21	0.834
Cluster extreme abuse											6.10	2.97	.07	2.06	0.040
Cluster high abuse											4.26	1.25	.12	3.40	0.001
Cluster moderate abuse											0.86	0.77	.04	1.13	0.260
Model summary															
R^2^ (adj. R^2^)	.030 (.026)					.186 (.176)					.204 (.190)				
Δ*R* ^2^	.030^**^					.156^**^					.018^**^				

Gender, reference category: men; Marital status, reference category: not married or long-term relationship; Expectedness, reference category: expected death; Loss of a child/partner/other family member/friend, reference category: loss of parent; Cluster extreme/high/moderate abuse, reference category: no/low abuse Cluster; Cluster extreme abuse, *a priori* defined outlier cluster.

^**^p <.01; ^*^p <.05.

## 4 Discussion

This study presents the first examination of the potential associations between PGD symptoms and child abuse in a representative German sample. The prevalence rates of child abuse observed in our sample were comparable to those reported in previous representative samples conducted in Germany. The present study identified a no/low, moderate, high and an *a priori* extreme abuse cluster, aligning with previous CTQ studies. In addition to variables related to the loss, the overall severity of child abuse and the high and *a priori* extreme abuse cluster were associated with higher PGD symptoms. Compared to German prevalence studies conducted in 2010 and 2016 (7), the proportion of individuals reporting child abuse seems to have increased on a descriptive level. The increase in prevalence is mainly due to individuals reporting severe to extreme abuse. The observed trend could be due to normal statistical fluctuations. However, the heightened attention and awareness of child abuse in society could lead to more people willing to report. All three subtypes are below the European average, with the largest difference found for emotional abuse, which is 14.4%. It is worth noting that our study is representative, in contrast to the meta-analysis where the European average was calculated based on non-clinical samples (3).

The co-occurrence of emotional abuse and physical abuse was reported in 3.3% of cases, making it the most frequently reported abuse in our study. In total, 6.1% of respondents indicated that they had experienced at least moderate-severe abuse in two or more subtypes. Previous studies have also shown that a significant number of individuals have experienced more than one subtype of child abuse (7, 9, 59). To account for the simultaneous occurrence of multiple child abuse types, we conducted a clustering procedure with the child abuse subtypes. The no/low abuse cluster exhibited the largest number of participants, with mean scores for all child abuse subtypes falling within the non-minimal level of the severity category by Häuser et al. (49). The mean scores of the child abuse subtypes of the moderate abuse cluster demonstrated minimal-moderate levels for emotional abuse and sexual abuse and non-minimal levels for physical abuse. Given the existence of disparate cutoffs for the subtypes, it would appear from **Figure 1** that only emotional abuse is elevated, rather than sexual abuse. However, with regard to sexual abuse, any score above 5 is already classified as minimal-moderate (49), as the effects on the individual are presumed to be more significant. The CTQ total score of the high abuse cluster is more than double those of the no/low abuse cluster. The mean scores of the emotional abuse subtype are classified as severe-extreme, for physical abuse as moderate-severe, and for sexual abuse as minimal-moderate. Other studies that employed the CTQ for clustering have also identified low, moderate, and high abuse clusters (20, 22, 23). The *a priori* extreme abuse cluster consists of nine individuals, and the mean scores of all child abuse subtypes were within the severe-extreme level (49). The separation of clusters by severity lends support to the hypothesis that focusing on the subtypes may not be sufficient (17) and that severity is a more important factor (18) for the analysis of child abuse. In all clusters identified by k-means analysis in our sample, sexual abuse was classified as minimal-moderate at most, according to the categories by Häuser et al. (49). However, the present sample comprises a total of 21 cases of severe-extreme sexual abuse. The exclusion of the outlier results in a 42.9% reduction in this category. The additional outlier cluster ensured the preservation of data information for further analyses. Other studies using CTQ clusters (17, 20–26) have not addressed the issue of removing outliers. However, it should be noted that these studies employed exploratory clustering methods. In contrast, k-means analysis, requires the number of clusters to be predetermined and is significantly more sensitive to the existence and skipping of outliers (52).

In our study, we observed that PGD symptoms were primarily associated with loss-related and child abuse-related factors, rather than demographic variables. While age, gender, and marital status were found to correlate significantly with PGD symptoms, they did not exhibit a significant influence in the final models. Although some studies suggest that being female is associated with increased PGD symptoms (e.g., 36, 37, 60, 61), this finding is not always consistent (e.g., 35, 38, 62). Despite the identification of an association between older age and PGD symptoms in certain studies (e.g., 28, 38), in our study age had no significant influence at any step of the analysis. Concerning marital status, there are studies that describe that being married is a protective factor (63), but most studies (e.g., 64) do not find any influence of being married on PGD symptoms. Our study found that being married or in a long-term relationship had a significant influence on the first two steps of the model, but not the last. Our findings, however, are consistent with the meta-analysis on predictors of PGD symptoms (33) that found no correlation with age and no correlation with marital status in the unadjusted analysis. The variables that had the greatest influence on the model were those related to the loss, such as time since loss, unexpectedness of death, and loss of a child or partner. These variables had similar effect sizes and together they explained 15.6% of the variance. Again, these findings are in line with recent meta-analytic results (33). In our model, time since loss had the greatest impact.

Among the variables related to child abuse, the overall severity of child abuse and the high abuse and *a priori* extreme abuse cluster contributed to higher PGD symptoms. The overall severity of child abuse was calculated as the sum of all child abuse subtypes. Therefore, elevated child abuse scores in our study may be indicative of the presence of comorbid child abuse subtypes and/or greater exposure to them. This finding leads us to expect an association between clusters with high child abuse values and increased PGD symptom severity. This conclusion is only partly supported by the results of the second regression analysis with the child abuse clusters. Individuals in the high abuse cluster experienced several child abuse subtypes to a moderate-extreme degree and exhibited elevated PGD symptoms. While individuals in the *a priori* extreme abuse cluster exhibited even higher child abuse values compared to those in the high abuse cluster, they showed a lower expression of PGD symptoms, although the impact still proved to be significant. The only study that already investigated the relationship between PGD and child abuse did not yield significant results (41). Importantly, this study focused on elderly individuals and used the Childhood Trauma Screener, which had poor internal consistency and did not distinguish between types of abuse and neglect. However, studies examining disorders that are often comorbid with PGD (depression, anxiety, and PTSD) have also demonstrated associations with child abuse (12, 13, 65). Interestingly, other studies that have clustered child abuse and examined its influence on mental disorders have also identified distinct child abuse intensity clusters rather than clusters according to discrete child abuse subtype clusters (23, 24, 66). In these studies clusters with high child abuse values were frequently associated with the presence of symptoms of disorders or their severity (17, 23, 24). An investigation of the distinctive subtypes of the two significant clusters in our study reveals that, although the mean values of all subtypes are elevated in the *a priori* extreme abuse cluster compared to the high abuse cluster, the sexual abuse mean value in the *a priori* extreme abuse cluster was more than three times higher than in the high abuse cluster. Therefore, the most distinctive difference between the clusters is to be found with respect to sexual abuse. Meta-analyses reveals primarily associations between emotional abuse and various mental health conditions, including depression, PTSD, and mental health in general (12, 13, 65). For sexual abuse lower associations than for emotional abuse were found with depression (13) and no association with mental health in general (12), but with anxiety (13). The lowest associations were found for physical abuse (12, 13). A similar trend could be suspected when considering the high abuse cluster of our study, where emotional abuse is at an extreme-severe level and physical abuse and sexual abuse are lower. This aligns with numbers of most countries where emotional abuse is more often reported than the other types of child abuse (3). Due to the limited sample size of nine participants, it is not possible to make a valid statement why the *a priori* extreme abuse cluster with highly elevated sexual abuse scores is less associated with PGD symptoms than the high abuse cluster. It is conceivable that, in addition to the intensity of child abuse, other mechanisms specifically associated with sexual abuse may contribute to the observed effects on PGD symptoms. One might assume that the severe experience of sexual abuse may lead to specific problems, e.g. in emotion regulation, that indirectly affect grief but are actually part of other symptoms, e.g. increased (complex)PTSD symptoms. However, given the *a priori* cluster’s limited sample size and the lack of data on comorbid disorders in the sample, this remains only a hypothesis.

It is also conceivable, however, that specifically the elevated emotional abuse values are a contributing factor. Spencer-Laitt et al. (67) propose that PGD is an emotional disorder that arises from early experiences of uncontrollability and unpredictability, as well as learned aversive reactivity to emotional experiences, which may create a vulnerability to PGD after loss. It is possible that uninvestigated third variables could explain the relationship between emotional abuse and PGD symptoms. Studies suggest that emotional abuse can lead to the development of cognitive and behavioral patterns (e.g., early maladaptive cognitive or emotional schemas, attachment styles), which can have an unfavorable effect on later mental health. The Young Schema Model posits that early maladaptive schemas develop during childhood when basic psychological needs, such as attachment, boundaries, autonomy, and self-direction, are not adequately met (68). A meta-analysis by Pilkington et al. (69) found a correlation between child abuse and early maladaptive schemas in adulthood, with emotional abuse showing stronger correlations and affecting more schemas than the other two types of child abuse. In another study (70) individuals who developed early maladaptive schemas exhibited more intense grief reactions and experienced greater difficulty in integrating the loss. The most highly correlated schemas were abandonment, vulnerability to harm or illness, and self-sacrifice (70). These three schemas were also related to emotional abuse. However, self-sacrifice only had a small correlation in the meta-analysis (69). It is reasonable to assume that early maladaptive schemas may enhance the comprehension of the association between emotional abuse and PGD symptoms. Further research is necessary.

### 4.1 Strengths and limitations

This study has several limitations. First, we used the PG13+9 (45) to examine PGD symptoms, which were not originally designed for PGD according to ICD-11 (30) or DSM-5-TR (31). Besides, we did not use diagnostic categories for our analyses, we used only the severity of PGD symptoms. Secondly, neglect was not assessed as a contributing factor alongside child abuse. However, a similar pattern emerged across studies (e.g., 17, 21, 22), whereby symptom severity was predominantly associated with child abuse clusters, rather than with neglect clusters. Third, it is possible that the survey method used could lead to an underrepresentation of individuals with a migration background (7). The majority of individuals (94%) in our sample held German citizenship, which is above the current average. It is important to note that holding German citizenship does not necessarily indicate Caucasian ethnicity. Given that the survey did not inquire about ethnicity and approximately 30% (71) of individuals with German citizenship have a migration background, the proportion of participants with a migration background is likely underestimated. Fourth, the investigation of child abuse was retrospective due to the cross-sectional study design. This type of data collection may underestimate the true prevalence of each child abuse type due to recall bias. Retrospective data collection is a common practice in child abuse studies, but it may lack reliability. According to a meta-analysis conducted by Baldwin et al. (72), nearly half of the participants failed to report child maltreatment when asked to recall it. Additionally, there is a lack of available data regarding the frequency or duration of child abuse, as well as the relationship between the offender and the deceased. Furthermore, the outliers have been placed in a separate cluster to prevent data loss. Consequently, the cluster is very small, which necessitates a cautious interpretation of the results of this *a priori* outlier cluster. Lastly, it should be noted that there is no data on comorbid psychiatric symptoms or cognitive or behavioral patterns that should be examined to understand the relationship between child abuse and PGD symptoms. Despite its limitations, our study has unique strengths. To our knowledge, this is the first study to examine the association between child abuse and PGD in adulthood in a representative sample of all ages. We used a valid measurement tool to investigate child abuse and its subtypes. We found comparable child abuse patterns, as observed in other studies. To our knowledge, no other study has explored the correlation between clusters of child abuse and PGD symptoms so far. The study highlights the relationship between child abuse and PGD symptoms, as well as gaps in research that need to be explored.

### 4.2 Further research and implications

Our study has left some questions unanswered, indicating the need for further research. This research should utilize survey instruments developed for PGD according to ICD-11 and DSM-5-TR. Additionally, comorbid disorders such as depression, PTSD, and anxiety disorders should be assessed to investigate their influence on the association between child abuse and PGD. Furthermore, it is important to identify and investigate possible mediating factors for child abuse and PGD symptoms. Research has shown that certain early maladaptive schemas may lead to increased grief reactions (70). These schemas may be developed as a result of childhood factors, such as emotional abuse. Therefore, investigating attachment styles, cognitive and behavioral patterns related to the connection of child abuse and PGD symptoms is necessary. A longitudinal study that assesses child abuse and outcomes, including grief, in adulthood and also examines possible moderating factors, such as behavioral and cognitive schemas, over a long period of time could provide a great deal of insight.

In conclusion, our study found that overall child abuse severity and being part of the high abuse or *a priori* extreme abuse cluster are risk factors for PGD symptom severity. Understanding that histories of child abuse increase the likelihood of heightened PGD symptoms is helpful in identifying individuals with an elevated risk for developing PGD after loss.

## Data Availability

The raw data supporting the conclusions of this article will be made available by the authors, without undue reservation.
